# Synthesis and Characterization of Novel Epoxy Geopolymer Hybrid Composites

**DOI:** 10.3390/ma6093943

**Published:** 2013-09-09

**Authors:** Giuseppina Roviello, Laura Ricciotti, Claudio Ferone, Francesco Colangelo, Raffaele Cioffi, Oreste Tarallo

**Affiliations:** 1 Department of Engineering, University of Naples Parthenope, INSTM Research Group Napoli Parthenope, Centro Direzionale Napoli, Isola C4, Naples 80143, Italy; E-Mails: laura.ricciotti@uniparthenope.it (L.R.); claudio.ferone@uniparthenope.it (C.F.); francesco.colangelo@uniparthenope.it (F.C.); raffaele.cioffi@uniparthenope.it (R.C.); 2 Department of Chemical Science, University of Naples Federico II, Complesso Universitario di Monte S. Angelo, via Cintia, Napoli 80126, Italy; E-Mail: oreste.tarallo@unina.it

**Keywords:** geopolymer, hybrid composites, epoxy resin, metakaolin

## Abstract

The preparation and the characterization of novel geopolymer-based hybrid composites are reported. These materials have been prepared through an innovative synthetic approach, based on a co-reticulation in mild conditions of commercial epoxy based organic resins and a metakaolin-based geopolymer inorganic matrix. This synthetic strategy allows the obtainment of a homogeneous dispersion of the organic particles in the inorganic matrix, up to 25% in weight of the resin. The materials obtained present significantly enhanced compressive strengths and toughness with respect to the neat geopolymer, suggesting their wide utilization for structural applications. A preliminary characterization of the porous materials obtained by removing the organic phase from the hybrid composites by means of heat treatments is also reported. Possible applications of these materials in the field of water purification, filtration, or as lightweight insulating materials are envisaged.

## 1. Introduction

Geopolymers are a class of synthetic inorganic aluminosilicate materials generally formed by reaction of an aluminosilicate with a silicate solution under strong alkaline conditions. In these conditions, free SiO_4_ and AlO_4_ tetrahedral units are generated and linked to yield polymeric precursors (–SiO_4_–AlO_4_–, or –SiO_4_–AlO_4_–SiO_4_–, or –SiO_4_–AlO_4_–SiO_4_–SiO_4_–) by sharing all oxygen atoms between two tetrahedral units, while water molecules are released [1,2].

Geopolymers have drawn attention because of their excellent mechanical properties, similar to those offered by the traditional Ordinary Portland Cement (OPC). Compared with OPC, geopolymers present many advantages, which make them a viable and an attractive alternative to traditional binders in numerous industrial settings [3]: (a) metakaolin (MK), the most common starting material, is generated by thermal activation of kaolinite clay at lower temperature than cement clinker synthesis. In addition, other common raw materials,* i.e.*, fly ashes and blast furnace slags, are inexpensive and environmental friendly industrial solid wastes [4,5,6,7,8,9,10,11,12]; (b) geopolymers develop higher strength in a shorter period at room temperature than OPC (about 70% of the 28 days compressive strength could be developed in 4 h after mixing) [13]; and (c) geopolymers show superior fire and acid resistance and much less shrinkage than OPC [14,15].

As far as environmental impacts are concerned, a lower depletion of natural resources is obtained by considering concretes and mortars made with different kinds of waste as artificial aggregates [16,17,18].

However, the brittle behavior and the low flexural strength of geopolymers, typical of a ceramic like material, usually affect their extensive applications as structural material [18].

To overcome this problem, geopolymer-based composites of various natures have been widely investigated [19]. In order to increase the strength and the toughness of geopolymer-based materials, much effort has been done on the incorporation of different organic polymers [9,20,21,22,23,24,25,26,27,28,29,30], such as polyvinyl acetate [13], polypropylene [26], or polyvinyl alcohol [27,28], or water-soluble organic polymers [29,30].

This kind of composites is usually obtained by blending the polymer with geopolymers, sometimes in presence of compatibilizers [31,32,33,34,35,36].

Very recently, we have described an innovative strategy for the chemical incorporation of an appreciable amount of organic polymer (up to 25% in weight) into a MK-based geopolymeric matrix [37]. This method is based on the concurrent* in situ* polycondensation reactions of the organic and inorganic components aiming at a closer interaction between the phases. This class of hybrid composites differs from those previously described because it is based on a chemical compatibility between the two phases and not on a simple blending.

By following this procedure, a good compatibility between the inorganic geopolymeric phase and the organic one was achieved by using synthetic epoxy resins whose composition was tailored to promote the formation of the greatest number of hydroxyl tails during the epoxy ring opening reaction. This makes the organic phase “temporarily hydrophilic” and increases its chemical compatibility with the aqueous inorganic phase [37]. The material we obtained, presented a homogeneous and stable in time dispersion of the organic microdomains into the inorganic phase, without addition of external additives, even at an appreciable concentration of resin. This constitutes an interesting but still not adequately investigated synthetic strategy, which affords to the obtainment of organic-inorganic hybrid composites, showing interesting mechanical properties and, in particular, a considerably reduced brittleness with respect to the geopolymeric matrix.

In the present paper we report the preparation and characterization of new geopolymeric hybrid composites obtained by the same synthetic approach but using commercial bi-component epoxy resins. The advantage in the use of commercial resins, with respect to the synthetic ones, lies in their moderate-cost availability and in their easy handling in massive amounts, thus allowing the preparation of composites even on a large scale [38]. This fact is of particular importance since these new materials present increased compressive strength respect to the pure geopolymer, suggesting their possible practical applications in the manufacture of lightweight, thermo-insulating, or thermo-resistant panels with improved toughness.

Furthermore, a preliminary characterization of the low density porous materials obtained by removing the organic phase by means of heat treatments is also reported. These materials could have possible applications in the field of water purification, filtration, or as lightweight insulating panels.

## 2. Results and Discussion

### 2.1. Synthetic Method

Epojet^®^ and EpojetLV^®^ are bi-component resins for injection. We have chosen these epoxy resins on the base of their chemical compositions and on their chemical-physical properties, that are similar to that of the synthetic resins we have previously designed [37] in order to obtain a good incorporation in the geopolymeric matrix.

The epoxy component of Epojet^®^ consists of diglycidyl ether hexanediol while the curing agent is 3,6-diazaoctane-1,8 diamine and m-xililen-diamine. In the case of EpojetLV^®^, instead, the epoxy component consists of the glycidylester of neodecanoic acid and xililen-diamine while the curing agent is 3-azapentane-1,5-diamine. Both resins contain also an aromatic amine that contributes to improve their thermal stability [39].

Hybrid composites Geo-Epojet and Geo-EpojetLV have been prepared incorporating the resin into the geopolymeric matrix suspension under mechanical stirring, when both polymerization reactions have not been completed yet.

This constitutes an interesting and still not sufficiently investigated strategy (it has been introduced by us only very recently) [37], which affords to the obtainment of homogeneous hybrid materials with increased performances with respect to the neat geopolymer.

**Scheme 1:**
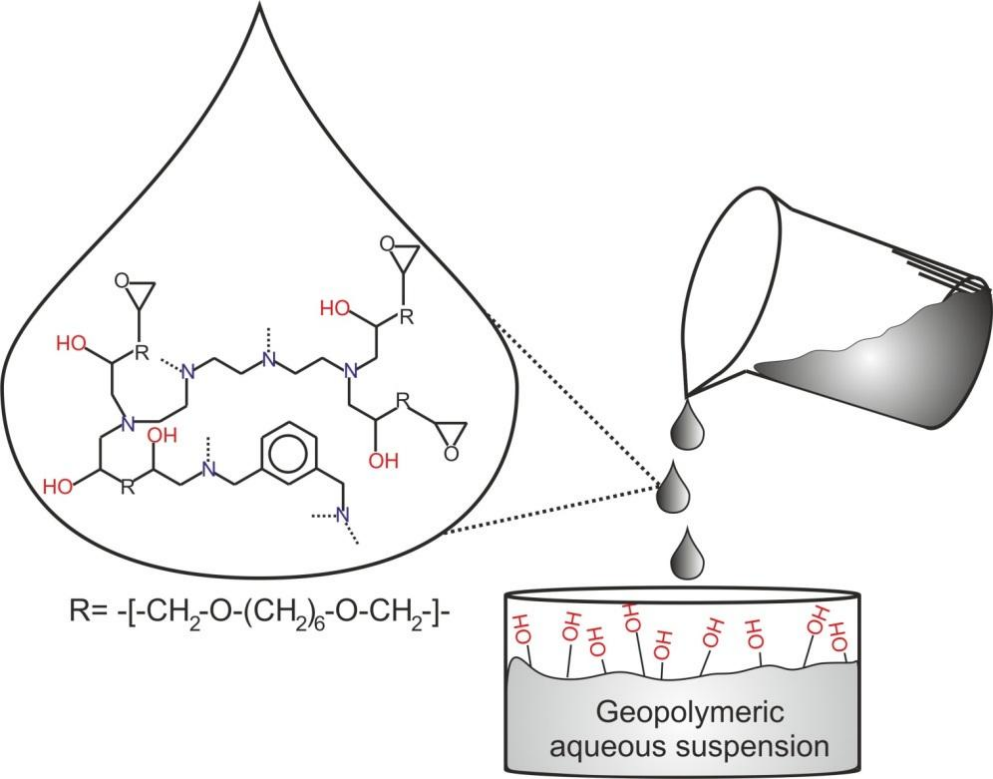
Schematic representation of the addition of the partly reticulated Epojet^®^ resin to the geopolymeric aqueous mixture.

The incorporation of the resins in the geopolymeric suspension, when both the polycondensation reactions were started but not yet completed, and the organic and inorganic phases were still easily workable, represents the pivotal step of the procedure. In this way, the large number of hydroxyl tails formed during the epoxy ring opening reaction makes the organic phase highly compatible with the aqueous inorganic one (see Scheme 1). It is worth noting that the addition of the unreacted components of the resins to the inorganic suspension produces phase segregation and, on the contrary, a late mixing of the two components (the cured organic resin and the geopolymer) strongly reduces their compatibility and the homogeneity of the dispersion of the two phases. For this reason, a careful realization of the synthetic procedure is essential to produce new materials with interesting properties.

In the preparation of the hybrid specimens, we have experimented different w/w ratios between the organic and the inorganic matrices, ranging from 5% to 30% w/w. However, the highest amount of resin that can be incorporated into the geopolymeric matrix without phase separation turned out to be 25% w/w as in the hybrid composite samples containing 30% w/w of organic resin an incipient phase segregation phenomenon has been observed.

Despite the complete characterization has been performed on all the specimens prepared with different w/w ratios between the organic and the inorganic matrices, in this paper only the results obtained in the case of the hybrid composites containing 80% weight geopolymer and 20% weight resin (Geo-Epojet20 and Geo-EpojetLV20), will be presented. This is because such a composition turned out to be the best compromise between the necessity of keeping a good compatibility of the two different phases and the need to produce a significant improvement on the mechanical properties of the inorganic matrix. As far as the specimens, with other compositions (with 5% and 10% w/w of epoxy resins), only the mechanical characterization will be shown as in that case the different compositions turned out to strongly affect the properties of the materials examined.

### 2.2. Characterization

#### 2.2.1. Thermal Analysis (TGA/DSC)

Simultaneous thermogravimetric and differential scanning calorimetry analyses were performed on the unmodified geopolymer, on the organic resins and of the hybrid composites, to compare their thermal behavior after curing at room temperature for seven days in 99% relative humidity conditions.

**Figure 1:**
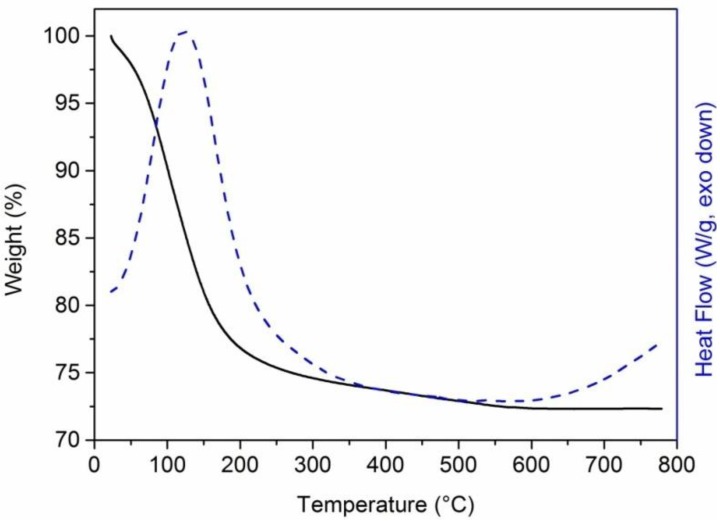
Thermal gravimetric analysis (TGA) (continuous line) and differential scanning calorimetry (DSC) (dashed line) curves of the neat geopolymer cured at room temperature for seven days in 99% relative humidity conditions.

Figure 1 shows the weight losses and the differential scanning calorimetry (DSC) thermograms for the unmodified geopolymers.

The weight loss starts at ≈30 °C with maximum weight loss temperature around 120–130 °C and is completed at ≈500 °C. This loss can be attributed to the removal of water molecules absorbed (up to ≈100 °C) or differently linked (up to ≈200 °C, free water in the pores; at higher temperatures, structural water and bound water in the nanopores) to the silicate molecules [40,41,42]. The overall weight loss is 28% and a residual of 72% remained at 800 °C. Correspondingly, the DSC curve of the geopolymer sample (dashed curve of Figure 1) shows a remarkable endothermic peak centered at 123 °C. It is worth pointing out that the variation of enthalpy associated to removal of water is in qualitative agreement with the weight loss of the specimen.

**Figure 2:**
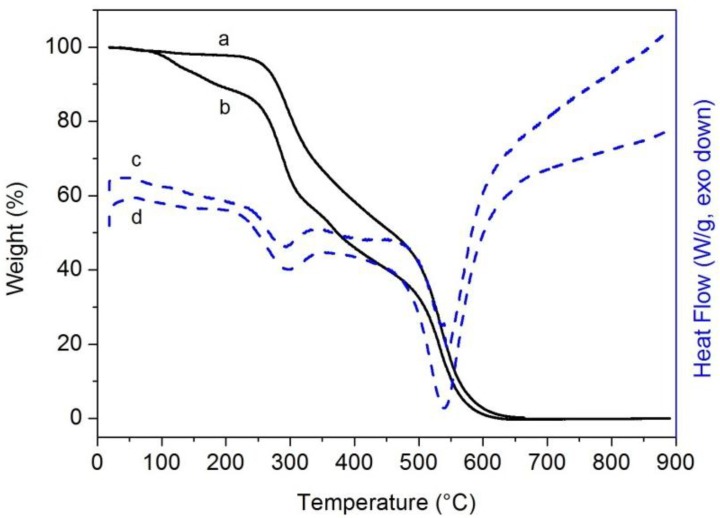
TGA (continuous lines) and DSC (dashed lines) curves of Epojet^®^ (a,c) and EpojetLV^®^ (b,d) resins cured at room temperature for seven days in 99% relative humidity conditions.

Figure 2 shows the weight loss and the thermograms for the unmodified Epojet^®^ and EpojetLV^®^ resins.

Epojet^®^ resin shows a degradation mechanism involving two main steps, while EpojetLV^®^ resin shows a three-steps one. Epojet^®^ is thermally stable up to about 250 °C. Above this temperature, a first degradation step that finishes at ≈480 °C is observed, resulting in a weight loss of 51%. The second degradation process is completed at about 650 °C and no combustion residual remains. As shown by the DSC curve, the two degradation steps are accompanied by two exothermic peaks; the first one centered at 294 °C and the second one at 538 °C.

EpojetLV^®^ is thermally stable up to about 100 °C. After this temperature, a first degradation step starts, corresponding to a weight loss of about 15%. The second degradation step starts at about 250 °C and finishes at about 500 °C, and corresponds to a further weight loss of ≈50%. The last degradation step, centered at ≈530 °C, is complete at temperatures around 650 °C with no combustion residual. Both steps are characterized by a high degradation rate. In addition, in this case the DSC curve shows two exothermic peaks centered at nearly the same temperatures found for Epojet^®^.

**Figure 3:**
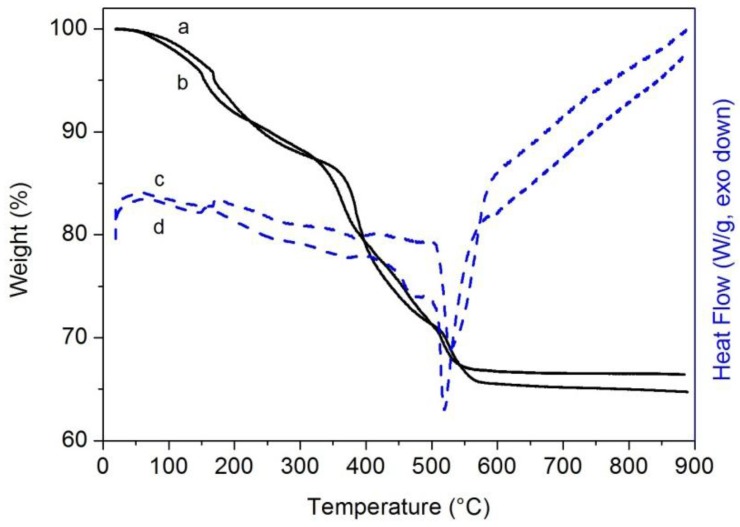
TGA (continuous lines) and DSC (dashed lines) curves of Geo-Epojet20 (a,c) and Geo-EpojetLV20 (b,d) specimens cured at room temperature for seven days in 99% relative humidity conditions.

As far as hybrid composites, Geo-Epojet20 and Geo-EpojetLV20, the thermogravimetric weight losses and the corresponding thermograms are shown in Figure 3.

In both cases the weight losses show a complex mechanism involving different steps: in particular, a first step, corresponding to a weight loss of ≈10%, is recorded up to ≈300 °C, while a second step, characterized by a complex path, is observed from ≈300 °C up to ≈600 °C and corresponds to a further weight loss equal to ≈20%. From the comparison of the TGA and DSC curves of the neat geopolymer and of the epoxy resins reported in Figure 1 and Figure 2, respectively, it is possible to associate the first degradation step mainly with the loss of water of the geopolymeric phase while the remaining one corresponds to the degradation of the dispersed organic phase. The combustion residual at 800 °C is about 65% in both cases.

Moreover, it is worth pointing out that the peak temperature of water loss for the composite is higher than that of the pure geopolymer: the polar groups of the resin probably interact with the water molecules delaying their evaporation.

Finally, it is also important to note that in both cases as also revealed by SEM images (Figure 7 in Section 2.2.4) a thermal treatment up to 800 °C produces a complete removal of the organic phase from the hybrid material.

Degradation temperatures and weight losses for all the studied systems are summarized in Table 1.

**Table 1:** Thermal properties of the neat geopolymer, pure epoxy resins, and hybrid composite specimens.

Systems	Weight loss starting temperature (°C)	Weight loss ending temperature (°C)	Residual at 800 °C (wt %)
Geopolymer	30	500	72
Epojet^®^ resin	250	650	0
EpojetLV^®^ resin	100	650	0
Geo-Epojet20	30	650	65
Geo-EpojetLV20	30	650	67

#### 2.2.2. Fourier Transform Infrared Spectroscopy (FT-IR) Analysis

**Figure 4:**
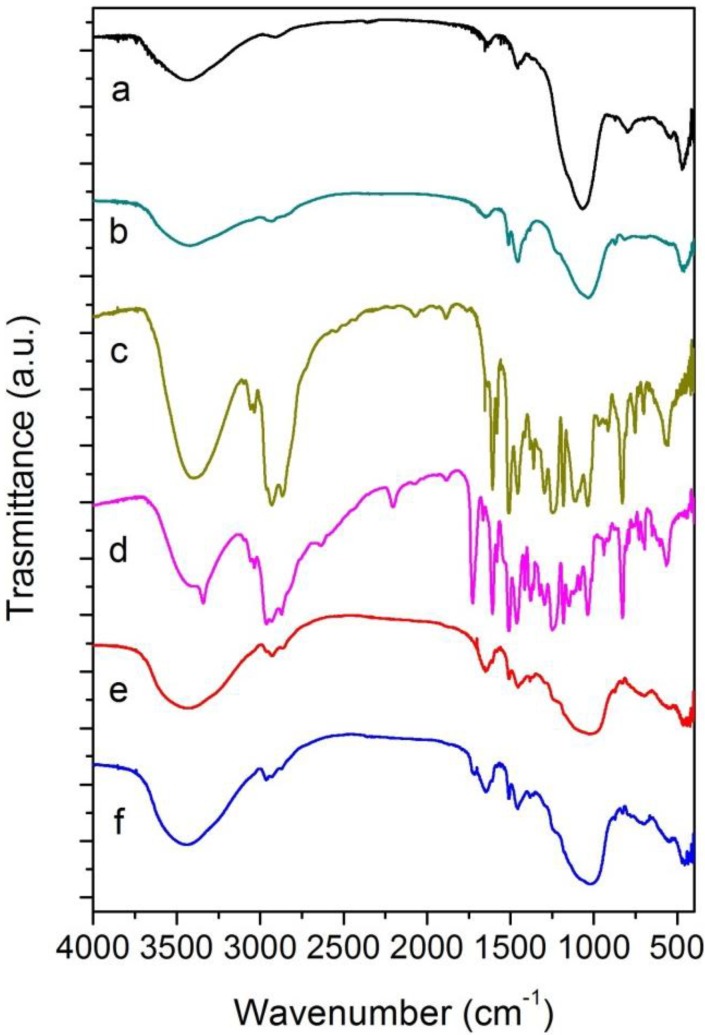
FT-IR spectra of (a) metakaolin; (b) geopolymer; (c) Epojet^®^; (d) EpojetLV^®^; (e) Geo-Epojet20; and (f) Geo-EpojetLV20.

The fourier transform infrared spectroscopy (FT-IR) spectra of the starting metakaolin, the geopolymer, the two organic resins, and of Geo-Epojet20 and Geo-EpojetLV20 hybrids are shown in Figure 4.

The FT-IR spectrum of the geopolymer (curve 4b) shows broad bands at about 3440 and 1630 cm^−1^ due to O–H stretching and bending modes of absorbed molecular water [43,44] and a strong band at about 1035 cm^−1^ due to Si–O stretching vibrations [44]. The lower wave number of this band than that of the corresponding one in the unreacted MK sample (centered at 1066 cm^−1^, curve 4a), indicates the condensation of Si–O tetrahedra in geopolymer. In particular, this shift toward a lower wavenumber may be attributed to the partial replacement of SiO_4_ tetrahedron by AlO_4_ tetrahedron, resulting in a change in the local chemical environment of Si–O bond [13,44]. Moreover, it is worth pointing out that the characteristic metakaolin Si–O–Al band at 810 cm^−1^ due to the six-coordinated Al(VI)–O stretching vibration, disappears in the geopolymer indicating a complete geopolymerization reaction [44]. Finally, the signal at about 460 cm^−1^ is due to Si–O bending vibration [44,45,46,47,48,49,50,51,52].

The FT-IR spectra of Epojet^®^ and EpojetLV^®^ (curve 4c,d, respectively) are very similar to each other. For both resins, the presence of the broad band at ≈3400 cm^−1^ is assigned to O–H stretching of hydroxyl group [48,53]. The signals in the wavenumber range 2968–2854 cm^−1^ and about 1460 cm^−1^ are due to –CH_2_– symmetric and asymmetric stretching and banding, respectively [48,53]. Moreover, signals located in the range 1300–1050 cm^−1^ can be assigned to C–N, C–C and C–O stretching [48,53]. In addition, it is possible to observe different bands that are due to aromatic rings: C–H stretching at about 3030 cm^−1^, C–H and C=C bending in the wavenumber ranges 860–680 and 1700–1500 cm^−1^ respectively. Finally, in the case of EpojetLV^®^ the additional band located at 1725 cm^−1^ which can be assigned to C=O stretching of ester group [48,53].

As far as the hybrid composites, their spectra (curve 4e,f, respectively) are characterized by the main bands of pure organic resins and of the inorganic geopolymer. In particular, the broad bands at about 3435 and 1630 cm^−1^ are due to O–H stretching and bending modes of absorbed molecular water while the band at 1040 cm^−1^ is due to Si–O stretching and that at about 460 cm^−1^ is due to Si–O bending vibration. Finally the bands in the region 800–600 cm^−1^ are associated to Si–O–Al vibrations [54,55,56,57].

#### 2.2.3. X-ray Diffraction Characterization

**Figure 5:**
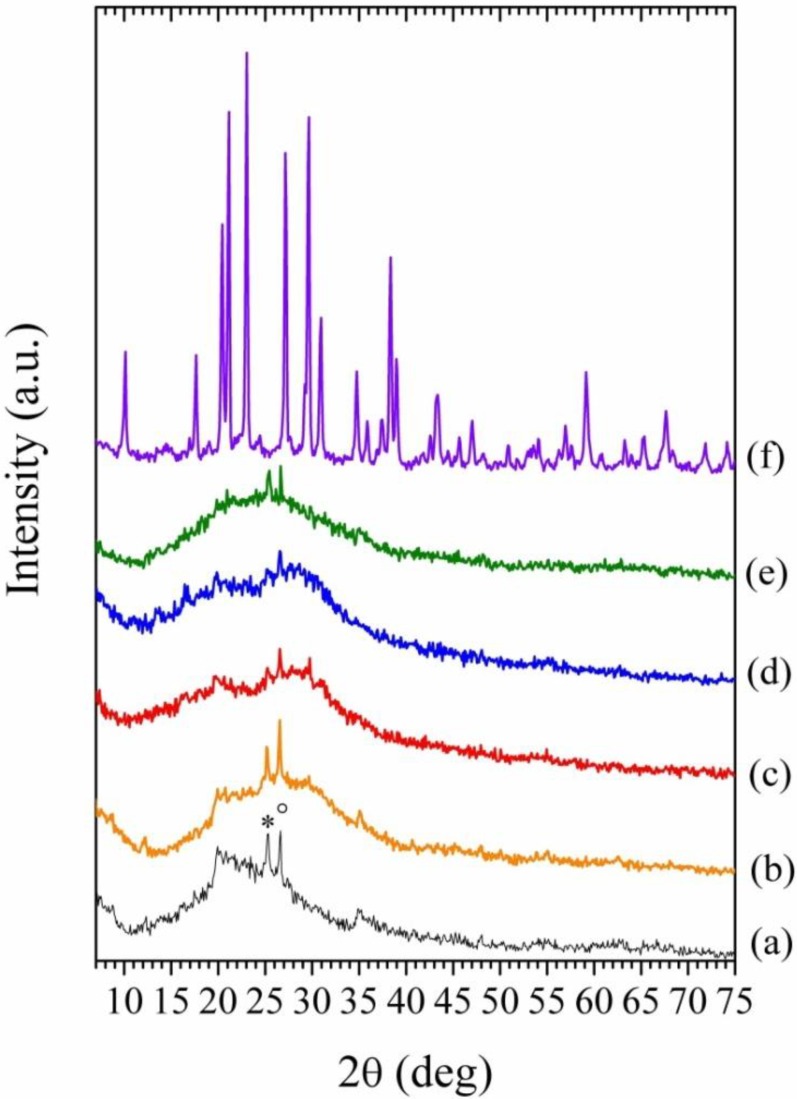
X-ray powder diffraction patterns of (a) metakaolin; (b) Geopolymer; (c) GeoEpojetLV20; (d) GeoEpojet20; (e) GeoEpojet20 after 12 h at 500 °C; and (f) GeoEpojet20 after 24 h at 800 °C. * = anatase; ° = quartz.

Figure 5 shows the X-ray powder diffraction patterns of the unreacted metakaolin (a); the neat geopolymer (b); the hybrid composites Geo-EpojetLV20 (c); Geo-Epojet20 (d); those of Geo-Epojet20 specimen after 12 h at 500 °C (e); and after 24 h at 800 °C (f).

**Figure 6:**
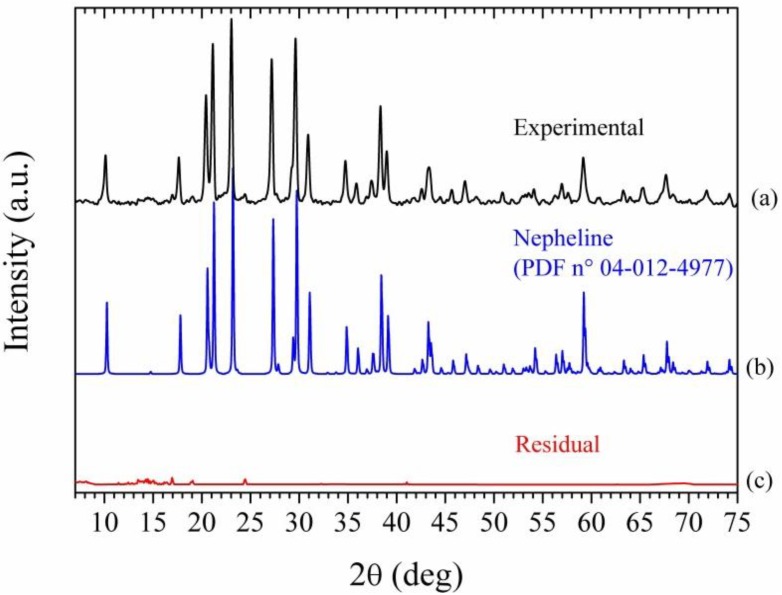
X-ray powder diffraction patterns of (a) GeoEpojet20 sample after 24 h at 800 °C; (b) nepheline (PDF n° 04-012-4977); and (c) residual.

As apparent, the diffraction pattern of the metakaolin specimen used for the preparation of the geopolymeric sample reveals that it is predominantly amorphous, being characterized by the presence of a large halo centered at 2θ ≈ 20°–25° and by some minor diffraction peaks revealing the presence of residual kaolinite (2θ ≈ 19°–21°) and small impurities probably represented by anatase (diffraction peak at 25.3°) and quartz (the strong peak at 27.5°). In line with what usually observed for samples prepared in similar conditions and with similar composition, the major feature of X-ray diffraction (XRD) powder diffraction patterns of the geopolymer (Figure 5b) is a largely featureless “hump” centered at approximately 27°–29°. In addition, both Geo-EpojetLV20 (Figure 5c) and Geo-Epojet20 (Figure 5d) specimens are amorphous. Geo-Epojet20 specimen keeps its amorphous structure upon heating up to 500 °C in air (Figure 5e) at a heating rate of 20 °C/min. Upon heating up to 800 °C instead, the sample is completely transformed into a crystalline phase (Figure 5f). This crystalline phase has been identified as nepheline (PDF n° 04-012-4977) (Figure 6). This result is in agreement with what already reported in the literature for heat-treated pure geopolymeric specimens of similar composition.

#### 2.2.4. Microstructural Analysis

**Figure 7:**
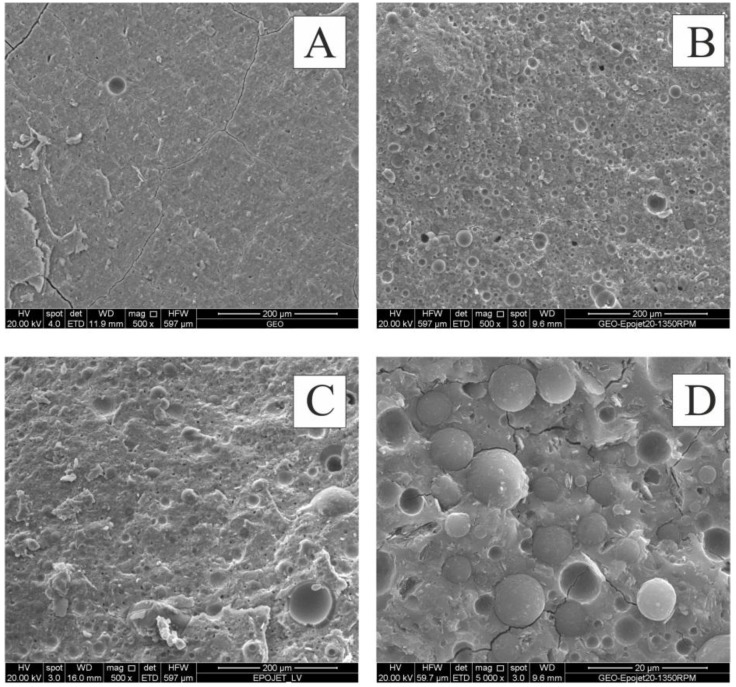
Scanning electron microscope (SEM) micrographs of (**A**) neat geopolymer; (**B**,**D**) GeoEpojet20; and (**C**) GeoEpojetLV20.

Figure 7 shows micrographs of freshly obtained fracture surfaces of geopolymer, Geo-Epojet20 and GeoEpojetLV20.

The geopolymer specimen (Figure 7A) shows a homogeneous amorphous structure indicating that the geopolymerization process has been successfully carried out. As far as hybrid composite specimens (Figure 7B–D), a very good homogeneity and uniformity of the micro dispersion of well-defined organic particles into the inorganic matrix is apparent. In particular, the dimension of the resin particles is in the range 1–10 μm. For all the hybrid composite specimens examined, no agglomerations phenomena were observed.

In all cases, the strict adhesion between the phases is evident because the particles of resin are scratched when the specimens are broken to prepare the scanning electron microscope (SEM) samples (see, for example, Figure 7D).

Moreover, comparing Figure 7B,C with 7A it can be easily seen that the number and the extension of the microcracks, that characterize the fracture surface of the geopolymer specimen, is strongly reduced by adding the organic resin. Therefore, in agreement with what observed by compressive strength tests (described in the following section), the resin seems to prevent the cracking growth and propagation improving the mechanical properties and enhancing the fracture toughness of the brittle inorganic matrix. As a matter of fact, a sort of crack deflection mechanism typical of particle reinforced ceramic matrix composites could be expected to occur [58,59].

**Figure 8:**
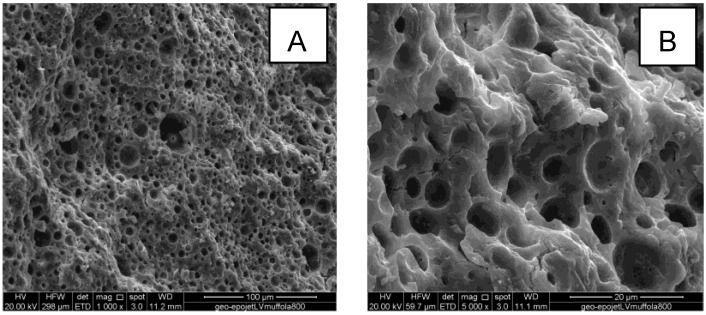
SEM micrographs of GeoEpojetLV20 sample kept for 24 h at 800 °C in air, at 1 × 10^3^ (**A**) and 5 × 10^3^ (**B**) amplifications.

Finally, Figure 8 shows the SEM image of a Geo-Epojet hybrid composite specimen after thermal treatment at 800 °C for 24 hours in air and whose X-ray diffractogram has been shown in Figure 5f. It is apparent that, in good agreement with the TGA results, the organic phase has been completely removed. In this way a highly porous material characterized by a disordered porous network with a continuous range of pore sizes, from nanometers to micrometers, has been obtained (see Figure 8).

**Figure 9:**
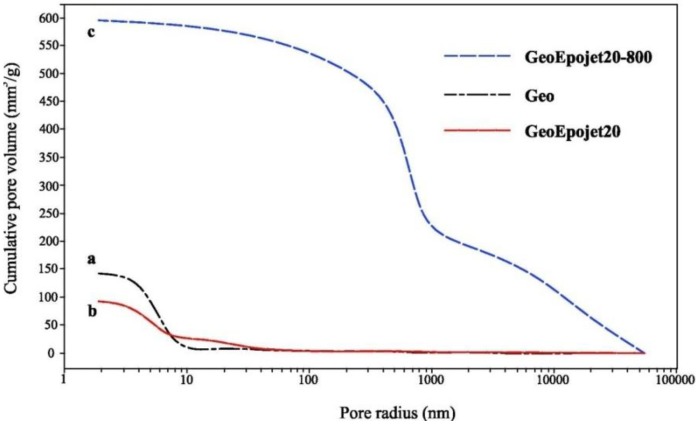
Cumulative pore volume* vs.* pore radius as obtained by mercury intrusion porosimetry analyses.

This nano- and macro-porosity has been confirmed by the mercury porosity data reported in Figure 9, where the cumulative volume of mercury intruded in mm^3^/g* versus* pore radius in nanometers has been reported for the unmodified geopolymer (a); the GEOEpojet20 (b); and the heat-treated GEOEpojet20 (c) specimens.

In particular, in the case of the neat geopolymer (Figure 9, curve a), it can be observed that only nanopores (with average diameters up to 10 nm) are observed. Probably these pores could be the result of pooling from regions of water that are generated in the polycondensation step [60]. Moreover, the total porosity expressed in terms of cumulative volume of mercury intruded is very low (≈150 mm^3^/g).

In the case of the Geo-Epojet hybrid composite (curve b), it can be observed that the use of resins slightly expands the pore radius distribution (pores with a diameters up to ≈40nm have been observed) but reduces the total pore volume in respect to those observed in the case of the neat geopolymer (≈100 mm^3^/g).

While metakaolin based geopolymers in general do not experience a great increase in pore volume after heating [61], in the case of the Geo-Epojet hybrid composite kept at 800 °C for 24 h, the average pore size significantly increases (curve c). This is clearly due to the removal of the organic resin particles. In fact, the heat-treated Geo-Epojet specimen is characterized by a broader distribution of pores, with sizes ranging from an average radius of nanometers (those already present in the as-prepared specimen) up to microns (that are the voids left by the organic resin particles). The total porosity expressed in terms of cumulative volume of mercury intruded was found to be significantly affected by the removal of the organic phase undergoing to an increment of 500% with respect to the non-heated sample.

These results suggest that the synthetic approach described in the present paper could represent an easy procedure for producing porous geopolymeric based materials characterized by an extended interconnected porosity (from nano- to macro-pores) where the average pore size could be controlled, for example, by changing experimental condition such as mixing time and velocity.

For these materials possible applications could be envisaged in the development of new inert and low cost scaffolds for the cell growing and the controlled release of active guest molecules with biological or pharmaceutical properties or as sieves for the filtration of particulate or as lightweight heat and acoustic insulating materials.

Moreover the impregnation of this porous materials with ion exchange resins or inorganic salts such as hydrous iron oxide, could lead to the production of adsorbents for the removal of contaminants such as arsenate [62,63] from water.

#### 2.2.5. Compressive Strength Determination

**Figure 10:**
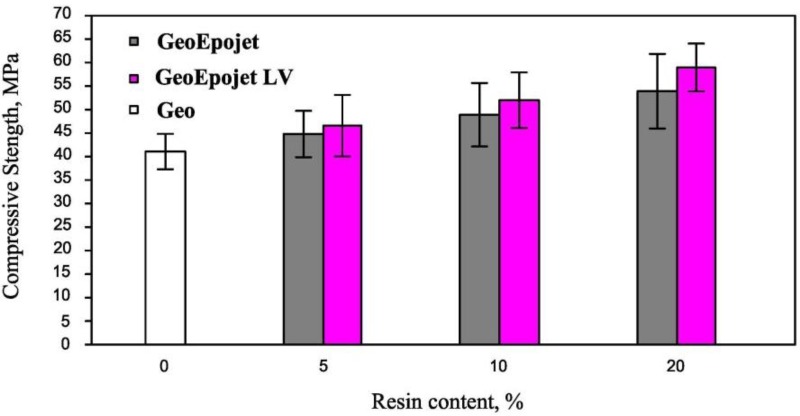
Compressive strength of the geopolymer, hybrid GeoEpojet and GeoEpojetLV specimens as function of resin content.

The compressive strengths of the GeoEpojet and GeoEpojetLV hybrid composite specimens; containing 5%, 10%, and 20% w/w of organic resin and cured for 28 days are shown in Figure 10. In the same Figure the compressive strength of the neat geopolymer sample; prepared and cured in the same conditions; is reported for comparison purposes.

The incorporation of the organic resin in the neat geopolymeric material affects the mechanical properties significantly, as the compressive strength increases with the resin content.

**Figure 11:**
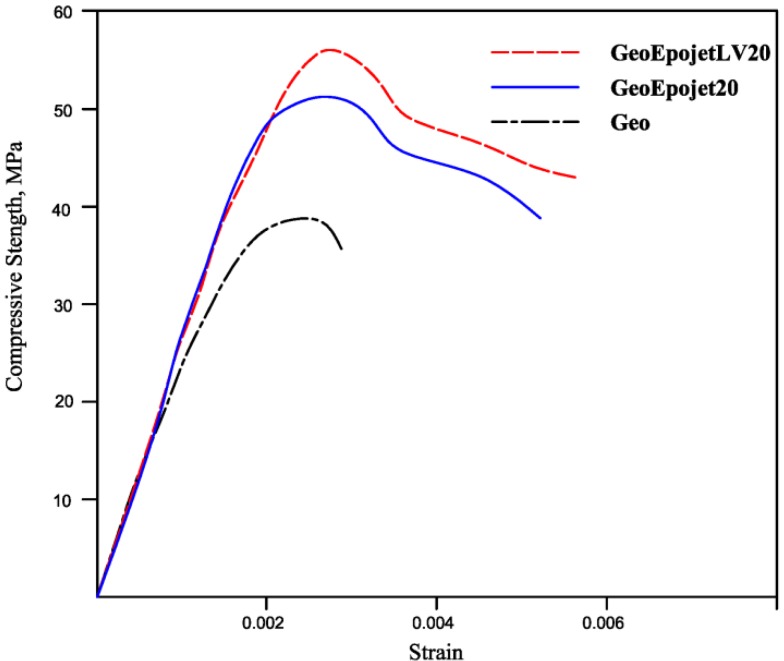
Stress-strain curves of the geopolymer, GeoEpojet20 and GeoEpojetLV20 specimens.

The compressive behavior of the materials studied in this work can be examined by plotting the complete stress–strain response (Figure 11). All the specimens were tested under uniaxial compression, by applying a vertical load gradually until they reached complete failure.

As already shown in the Figure 10, hybrid specimens have higher strength than the neat geopolymer one. The shape of the stress–strain curves of the hybrid material tested can be characterized with a linear elastic response up to about 40%–50% of its ultimate load carrying capacity. Close to the peak load, some cracks started appearing on the surface of the specimen. An examination of the stress–strain diagrams indicates that the hybrid specimens behave differently respect to the neat geopolymer. In fact, the stress-strain curve related to composite specimens continues at high stress values after the maximum and fracture happens at high strain values. On the other hand, the neat geopolymer sample fails at a strain value close to that of the maximum.

The peak stress (compressive strength value) and the corresponding value of strain, the ultimate strain (maximum strain of the samples) and the corresponding value of strain obtained are reported in Table 2.

**Table 2:** Ultimate strain, compressive strength at ultimate strain, strain at peak stress and unconfined compressive strength of the neat geopolymer and of the hybrid composite specimens.

Systems	ε_ult_^a^	σ_ult_^b ^(MPa)	ε_sps_^c^	σ_sps_^d ^(MPa)
Geopolymer	0.0029	35.68	0.0024	38.79
GeoEpojet20	0.0052	38.82	0.0027	51.27
GeoEpojetLV20	0.0056	43.02	0.0027	56.06

Notes: ^a^ Ultimate strain; ^b^ Compressive strength at ultimate strain; ^c^ Strain at peak stress; ^d^ Unconfined compressive strength.

Up to now, there are no structural codes for geopolymers. According to several codes [64,65], the ultimate strain is 3.5‰ for conventional concrete; for this value the compressive stress value is 60% of cylinder compressive strength at 28 days.

Figure 11 and Table 2 show that the ultimate strains of the composite specimens are higher than the strains considered by code for conventional concrete.

Even if the modulus of elasticity is similar for both hybrids, the use of GeoepojetLV allows to reach higher value of peak stress and ultimate strain.

As seen, the hybrid composite specimens show progressive fracture behavior rather than a brittle one and consequently require higher fracture energy than the neat geopolymer one. This behavior may be likely explained by considering the propagations of cracks proceeding in a progressive and controlled way with increasing strain [34] owing to the presence of the resin phase. The organic phase may play a dual role: on one side it could absorb part of the load by plastic deformation; on the other side it could perform a toughening effect by a typical crack deviation mechanism [58,59,66], as evidenced also by SEM observations (see Figure 7).

## 3. Experimental Section

### 3.1. Materials

The epoxy resins used in this paper, Epojet^®^ and EpojetLV^®^, were purchased by Mapei S.p.A (Milan, Italy). Sodium hydroxide was purchased by Aldrich. Metakaolin, provided by Neuchem S.r.l. (Milan, Italy), has the following composition: Al_2_O_3_ 41.90 wt %; SiO_2_ 52.90 wt %; K_2_O 0.77 wt %; Fe_2_O_3_ 1.60 wt %; TiO_2_ 1.80 wt %; MgO 0.19 wt %; CaO 0.17 wt %. The sodium silicate solution was supplied by Prochin Italia S.r.l. with the composition: SiO_2_ 27.40 wt %, Na_2_O 8.15 wt % and H_2_O 64.45 wt %.

### 3.2. Analytical Methods

Thermogravimetric (TGA) and differential scanning calorimetry (DSC) analyses were performed by a TA Instrument SDT2960 simultaneous DSC-TGA (TA Instrument, New Castle, DE, USA). The thermographs were obtained at a heating rate of 10 °C/min using ≈10 mg of the powdered sample under air flow.

Heat treatments on hybrid composite specimens have been performed by using a Neytech 15P muffle (Cole-Parmer Instrument Company, Vernon Hills, IL, USA). The samples were heated from room temperature to 500 °C and 800 °C, in air, at a heating rate of 20 °C/min.

FT-IR measurements were performed using a Jasco FT/IR-430 spectrometer (Jasco Corparation, Hachioji-shi, Japan). As far as the geopolymer and hybrid composites specimens, the experiments were carried out by using KBr discs in which a few milligrams of the already cured specimens was dispersed. Otherwise, the organic resins were analyzed by using free standing thin films.

Wide-angle X-ray diffraction patterns were obtained at room temperature with nickel-filtered Cu Kα radiation with an automatic Philips powder diffractometer operating in the θ/2θ Bragg-Brentano geometry using specimen holders of thickness equal to 2 mm. The phase recognition was carried out by using the PDF-4+ 2012 (International Centre for Diffraction Data^®^) database and the HighScore Plus (PANalytical) software.

The pore size distribution and the density of the specimens were determined by means of mercury intrusion porosimetry (MIP) using Thermo Pascal 140 and 440 (Thermo Scientific, Milan, Italy).

SEM analysis was carried out by means of a FEI Quanta 200 FEG microscope (FEI, Hillsboro, OH, USA).

The compressive strength of geopolymer and hybrid specimens was measured by testing cubic concrete specimens (40 × 40 × 40 mm^3^) in a Controls MCC8 compression-testing machine (2000 kN) (Controls, Cernusco s/N. (Mi), Italy). The compressive strength was calculated from the failure load divided by the cross-sectional area resisting the load and reported in MPa. The values reported are the averages of the three compressions strength values.

### 3.3. Specimen Preparation

Preparation of resins: Epojet^®^ is a two-component epoxy adhesive for injection, which, after the mixing, takes the aspect of a low viscosity liquid. It is usable for 40 min at room temperature. EpojetLV^®^ is a highly low-viscosity two-component epoxy adhesive for injection in microcracks which, after the mixing becomes like a fluid liquid. Respect to Epojet^®^, EpojetLV^®^ takes a longer time to harden, since it is usable for ≈70 min.

Epojet^®^ and EpojetLV^®^ resins were obtained by mixing their components in 4:1 ratio in weight as specified in the technical data sheet supplied by the manufacturer [39].

Preparation of geopolymer: The alkaline activating solution was prepared by dissolving solid sodium hydroxide into the sodium silicate solution. The solution was then allowed to equilibrate and cool for 24 h. The composition of the solution can be expressed as Na_2_O 1.4SiO_2_ 10.5H_2_O. Then metakaolin was incorporated to the activating solution with a liquid to solid ratio of 1.4:1 by weight, and mixed by a mechanical mixer for 10 min at 800 rpm. The composition of the whole geopolymeric system can be expressed as Al_2_O_3_ 3.5SiO_2_ 1.0Na_2_O 10.5H_2_O, assuming that geopolymerization occurred at 100%.

Preparation of hybrid composites: Before being added to the geopolymeric mixture, Epojet^®^ and EpojetLV^®^ were cured at room temperature for 10 and for 60 min, respectively. Both the resins were added when they were still easily workable and long before their complete crosslinking and hardening (that takes place in about 5–7 hours at 23 °C).

Different specimens containing up to 20% w/w of resin were prepared by adding the resins to the freshly-prepared geopolymeric suspension, and quickly incorporated by mechanical mixing (5 min at 1350 rpm). In this way, GEO-Epojet5, GEO-Epojet10 and GEO-Epojet20 (in which the Epojet^®^ resin was 5%, 10%, and 20% in weight respectively) and GEO-EpojetLV5, GEO-EpojetLV10 and GEO-EpojetLV20 (in which the EpojetLV^®^ resin was 5%, 10%, and 20% in weight respectively) were obtained. All the hybrid composites presented a homogeneous aspect and started solidifying in few minutes.

Curing treatments: All the specimens, as soon as prepared, were poured in cubic molds and cured in >95% relative humidity conditions at room temperature for seven days (the samples used for the mechanical tests were left further 21 days in air; room temperature corresponds to about 25 °C) [67]. The evaporation of water was prevented by sealing the top of the molds with a thin plastic layer during storage as well as during the curing stage.

## 4. Conclusions

Through an innovative synthetic approach based on a co-reticulation in mild conditions of commercial epoxy based organic resins and a MK-based geopolymer inorganic matrix, new hybrid organic-inorganic materials were prepared. A high compatibility between the organic and inorganic phases, even at appreciable concentration of resin (25% w/w), was realized up to micrometric level. A good and homogeneous dispersion (without the formation of agglomerates) of the organic particles was achieved.

These new materials show good mechanical properties: in particular, in respect to the neat geopolymer, they present significantly enhanced compressive strengths and toughness.

By comparing our novel hybrid organic-inorganic composite materials with traditional geopolymer it is evident that: (i) the compressive strength of our material is significantly higher than that of the neat geopolymer; (ii) hybrid materials show higher deformation before cracking. Moreover, it is worth pointing out that in the synthetic procedure adopted, the use of solvents is completely avoided. From an environmental point of view, this means that it is possible, with a simple procedure and using inexpensive and easily available reagents to obtain a material which, respect to the common geopolymer, allows to save materials, to use smaller section for the same load condition, to reduce the number of cracks obtaining more durability and, thus, a longer service life. Moreover, the possible use of wastes coming from different activities (mining, metallurgic, municipal, construction, and demolition) instead of a raw material (metakaolin) for the obtainment of hybrid geopolimeric materials with our new synthetic approach could further reduce the environmental impact of the material we have studied.

Finally, the removal of the organic phase from the hybrid composite specimens through heat treatments has allowed us producing very promising highly porous material characterized by a disordered porous network with a continuous range of pore sizes, from nanometers to micrometers, suggesting possible applications in the field of water purification, filtration, or as lightweight insulating materials.

In order to better assess their possible technological applications, further studies both on the hybrid composites presented in the present paper and on the porous materials that can be obtained from them are in progress.
